# Microwaves Versus Combined Microwaves and Fractional Carbon Dioxide Laser in the Treatment of Postpartum Abdominal Laxity Among Filipino Patients in a Tertiary Hospital: A Randomized, Prospective, Assessor‐Blinded, Simultaneous Split‐Abdomen Trial

**DOI:** 10.1111/jocd.70237

**Published:** 2025-05-15

**Authors:** Jonnie Rose Louise R. Wee, Zharlah Gulmatico‐Flores, Daisy King‐Ismael

**Affiliations:** ^1^ Department of Dermatology Jose R. Reyes Memorial Medical Center Manila Philippines

**Keywords:** abdomen, fractional carbon dioxide laser, microwaves, postpartum, skin laxity

## Abstract

**Background:**

Postpartum abdominal laxity is a growing concern for women. Noninvasive options like microwave technology and fractional carbon dioxide (CO_2_) laser show promise, but their combined efficacy and safety require further investigation.

**Aim:**

To compare the efficacy and safety of microwaves versus combined microwaves and fractional CO_2_ laser in the treatment of postpartum abdominal laxity among Filipino patients.

**Patients/Methods:**

Thirty‐two patients with Fitzpatrick skin types III–V and postpartum abdominal laxity received three microwave sessions, with one side randomly assigned an additional fractional carbon dioxide laser session (designated as Side B, while the other as Side A). Global aesthetic improvement scale (GAIS) scores and patient satisfaction (PS) scores were determined at every follow‐up. Baseline and completion anthropometric measurements were taken, and adverse effects were recorded.

**Results:**

Significant improvements in GAIS and PS scores were noted for both sides across all sessions (*p* < 0.001), with side B showing superior scores post‐CO_2_ laser (*p* < 0.001). A moderate correlation between metabolic equivalent (METs) scores and GAIS scores (*p* = 0.413, *p* = 0.019) indicated that higher levels of physical activity were associated with higher GAIS scores. These improvements were attributed to epidermal thickening and dermal collagen and elastin remodeling, the latter seen histologically in a representative patient. Adverse effects were mild and noted only with CO_2_ laser.

**Conclusions:**

The combined use of the microwave system and fractional CO_2_ laser is safe and well tolerated and is superior to microwaves alone in the treatment of postpartum abdominal laxity.

## 1 Introduction

Pregnancy exerts significant effects in a woman's body [1]. In addition to a variety of hormonal, immunologic, and metabolic changes, the increasing abdominal volume from the enlarging uterus and weight gain underlie the cutaneous changes that may concern patients [1]. Many of these changes persist beyond pregnancy [2], which have been proven to negatively impact the body image, self‐esteem, and mental well‐being of women worldwide [3].

It is common for the postpartum patient to suffer from an increased skin laxity in the abdomen, often coexisting with an increased abdominal girth and striae distensae, even after losing the weight gained from pregnancy [2]. Postpartum abdominal laxity manifests as folds of loose excess skin that can develop at the waist, around the umbilicus, overhanging a cesarean section scar, or the hips [2]. While surgical correction, called abdominoplasty or “tummy tuck,” produces the most definitive results, it also requires significant recovery time. It carries several risks and sequelae such as infection, bleeding, and scarring [2]. The growing interest of patients to minimize downtime, procedural risks, and sequelae to address this concern caused the advent of noninvasive modalities to meet this demand, such as devices that use lasers, radiofrequency, and ultrasound waves to induce chemical and physiologic changes in the dermis to reduce skin laxity [4].

Microwave systems have been developed as an emerging treatment to reduce localized fat, minimize cellulite, and tighten skin. They work by delivering microwave energy to heat and destroy the target tissue [5]. Although this technology has already been in clinical use for body remodeling for the last 5 years, limited studies exist in the English literature regarding its efficacy, with most of the evidence focusing on its ability to reduce localized adiposity [5, 6, 7]. No studies have been done to evaluate its effect on the dermis.

Fractional carbon dioxide (CO_2_) laser is one of the first noninvasive techniques developed to tighten skin, inducing neocollagenesis and dermal remodeling to improve skin texture [8]. Although many studies have shown that fractional CO_2_ laser treatment is effective in improving various conditions such as acne scars, hypertrophic scars, and pigmentary changes, there is limited evidence specifically on its skin tightening effect, especially on laxity outside the face and neck, particularly the abdomen.

This study is the first to compare the efficacy and safety of the combined use of microwave technology and fractional CO_2_ laser versus microwave technology alone in the treatment of postpartum abdominal skin laxity, providing dermatologists essential insight into optimizing noninvasive management strategies for this prevalent concern.

## 2 Methodology

### 2.1 Study Design

This is a comparative, randomized, prospective, assessor‐blinded, split‐abdomen experimental study conducted at a tertiary care hospital from December 2023 to August 2024 after approval from the Institutional Review Board (IRB).

### 2.2 Participant Selection

Thirty‐two Filipino women at least 18 years old with Fitzpatrick skin types (FST) III–V who were at least 6 months (if delivered vaginally) or 2 years (if delivered via cesarean section) postpartum with visible wrinkling and/or sagging of the skin of the periumbilical and lower abdominal area were recruited in this study through nonprobability purposive sampling. All enrolled participants could understand English or Filipino instructions and signed a written informed consent.

Exclusion criteria include pregnancy, breastfeeding, or planning pregnancy within the next 4 months; BMI over 30; recent weight fluctuations; abdominal surgery, procedures with energy‐based devices, topical or injectable treatments, or oral intake of vitamin A derivatives, steroids, or anticoagulants within the last 6 months; history of herpes, hepatitis B or C, hypertrophic or keloid scars, transplants, active or past phlebitis, thrombophlebitis, connective tissue or uncontrolled systemic diseases; those with implants, pacemakers, allergies to lidocaine, prilocaine, or paraffin oil, or sensitivity to microwaves/lasers; smoking history; striae rubra or nigra; diastasis recti, and active abdominal dermatosis.

There were no attritions during the duration of this study.

### 2.3 Intervention

#### 2.3.1 Initial Evaluation

Clinical and demographic characteristics of subjects, including age, parity, months postdelivery, physical activity (in metabolic equivalents [METs] per day), FST, and type of delivery, as well as baseline measurements, were gathered using a researcher‐administered Data S1. Subjects received a printed copy of pre‐ and posttreatment instructions and were then scheduled for their first microwave treatment.

#### 2.3.2 Microwave Treatment Procedure

The abdomen was cleansed with mild soap and water and dried with a paper towel. A 15 cm × 15 cm treatment area was marked on each side of the abdomen, targeting visibly sagging and wrinkled areas on the periumbilical and lower anterior abdomen. Five milliliters of pure paraffin oil was applied to the treatment area. The shallow handpiece of the ONDA Coolwaves system (DEKA, Florence, Italy) was used with standardized manufacturer settings for skin tightening (120 W, 60000 J, 8 min 20 s) and a skin cooling temperature of 5°C. The handpiece was held perpendicular to the skin and moved in a smooth, continuous linear motion with light, even pressure. After treatment, the area was massaged for 10 s before the residual paraffin oil was wiped off. The procedure was repeated on the opposite side, and posttreatment instructions were reinforced. Subjects underwent three microwave treatments, spaced 1 month apart.

#### 2.3.3 Fractional CO_2_ Laser Treatment Procedure

One month after the last microwave treatment, an additional fractional CO_2_ laser treatment was randomly allocated to one 15 × 15 cm side of the abdomen by drawing lots (designated as Side B, while the other side as Side A). Side B was cleansed with mild soap and water, and a topical anesthetic (lidocaine 2.5% and prilocaine 2.5% cream) was applied for 60 min under occlusion with cling wrap, then washed off and dried. Participants were given specialized laser goggles to protect their eyes during the procedure. The treatment area, the same as that previously treated with microwaves, was marked with a skin marker. Using the fractional handpiece, the area underwent 10 600‐nm fractional CO_2_ laser (SmaXelAbdominal laxity, commonly referred to as sagging skin; IDS, Korea) with parameters based on the manufacturer's skin‐tightening protocol (spot size: 15 × 15 mm; pulse energy/dot: 36 mJ; pulse duration: ultra pulse [90 μs–900 μs]; depth: 500 μm; density: 13 spots/cm^2^; square pattern). The area received one pass, with forced air cooling to minimize discomfort. Afterward, a cold compress was applied for 5–10 min, followed by topical mupirocin 2% ointment. The contralateral side received no treatment, and posttreatment instructions were reinforced. Subjects returned after 1 month for a final follow‐up evaluation.

### 2.4 Outcome Evaluation

Clinical photographs of the abdomen were taken with a 100‐megapixel camera (realme 11 Pro 5G, realme) with fixed aperture, ISO, and shutter speed settings, against a blue background in a well‐lit room under uniform body positions, camera angle, height, distance, and lighting, with anterior, 45‐degree and 90‐degree lateral views taken at a uniform distance from the wall as guided by fixed floor markings [9].

Using these photos, improvement of skin laxity was rated on each hemiabdomen using the Global Aesthetic Improvement Scale (GAIS) [10] (Table 1) by three blinded board‐certified fellows of the Philippine Dermatological Society.

**TABLE 1:** Global aesthetic improvement scale.

Score	Degree	Description
0	Unchanged or Worsened	The appearance substantially remains the same or worsened compared with the original condition
1	Improved	Improvement of the appearance, better compared with the initial condition, but a touch‐up is advised
2	Much improved	Marked improvement of the appearance, but not completely optimal
3	Very much improved	Excellent corrective result

GAIS scores were obtained 1 month after every treatment session on each hemiabdomen. A score of 1 and above was considered a significant improvement from baseline.

Subjects' satisfaction was also graded on each hemiabdomen using the Patient Satisfaction (PS) score (Table 2) 1 month after every treatment session. A score of 2 and above was considered significant satisfaction with the improvement from the baseline.

**TABLE 2:** Patient satisfaction score.

Patient satisfaction score	
0	Not satisfied
1	Slightly satisfied
2	Satisfied
3	Very satisfied
4	Extremely satisfied

Waist circumference (WC) and waist‐to‐hip ratio (WHR), measured according to WHO expert committee guidelines [11], and body mass index (BMI), computed from height and weight, were determined at the initial and completion consult.

After obtaining informed consent, one patient volunteered to undergo 4‐mm skin punch biopsies on both sides of the abdomen at baseline and completion follow‐up. All biopsy specimens were fully submerged in 10% buffered formalin and processed into paraffin blocks before being cut into tissue sections. All sections were stained with hematoxylin and eosin, Masson Trichrome for examination of the epidermis and dermal collagen, and Verhoeff–Van Gieson for staining elastic fibers.

Data on adverse effects, for example, the sign or symptom, severity (rated from 1 to 10), onset, duration, and management done, were also obtained immediately and 1 month after treatment sessions.

### 2.5 Statistical Analysis

#### 2.5.1 Validation in Repeatability

Prior to this trial, the GAIS underwent validation in repeatability through evaluation of intra‐ and interobserver correlation [12]. This was done with two board‐certified dermatologists, other than those who took part in the actual scoring of data collection, who evaluated 20 sets of before‐and‐after photographs of the abdomen. Cohen k was calculated to be 0.612 with significance (*p* = 0.006). Therefore, the agreement between the two dermatologists was substantial, and the instrument had interobserver reliability and is repeatable, making it appropriate for the study.

#### 2.5.2 Sample Size Calculation

This study's sample size calculation utilized data from Bonan and Verdelli's pilot study [13] on treating postpartum abdominal laxity. Using the higher standard deviation of 8.4 cm from baseline waist circumference measurements, a 95% confidence interval, and a 5‐cm margin of error, the minimum required sample size was 25, ensuring at least 80% power. This study enrolled 32 participants with 88% power.

#### 2.5.3 Data Analysis and Statistical Considerations

Data analysis employed descriptive statistics (mean, median, standard deviation, interquartile range [IQR], minimum and maximum value) for numerical variables and frequency distribution/percentage for categorical variables. The GAIS and PS Scores were presented using the median and IQR. Paired sample *t*‐tests compared pre‐ and posttreatment differences for BMI, WC, and WHR since normality assumptions were met. The Wilcoxon signed‐rank test also compared median GAIS and PS scores from baseline across successive treatment sessions and between both sides. Spearman's correlation assessed associations between patient profile, WHR, A‐GAIS scores, and patient satisfaction scores. Group comparisons (e.g., vaginal vs. abdominal delivery) utilized the point‐biserial correlation for GAIS, satisfaction scores, and WHR. All analyses used a 95% confidence interval and a 0.05 significance level. Data were analyzed using SPSS version 26 after entry into Microsoft Excel.

## 3 Results

Table 3 shows the clinical and demographic characteristics of 32 participants. Ages ranged from 24 to 67 years (mean = 40.78, SD = 10.78). Median parity was 2.03 (IQR = 1.12). Time since the last delivery ranged from 9 to 412 months (mean = 127.94, SD = 102.29). MET scores ranged from 25.22 to 77.96 (mean = 35.38, SD = 10.94). FSTs were predominantly Type IV (53.13%), followed by Type III (37.50%) and Type V (9.38%). Most participants had vaginal deliveries (56.25%), with 43.75% having cesarean sections.

**TABLE 3:** Demographic and clinical characteristics of patients (*N* = 32).

Patient characteristics	M (SD) or *n*	Range or %
Age (years)	40.78 (10.78)	24–67
Parity	2.03 (1.12)	1–6
Months postdelivery	127.94 (102.29)	9–412
METs scores of physical activity	35.38 (10.94)	25.22–77.96
FST		
III	12	37.50
IV	17	53.13
V	3	9.38
Type of delivery		
Vaginal	18	56.25
Abdominal (CS)	14	43.75

There was a nonsignificant reduction in BMI from baseline (M = 25.14, SD = 3.21) to posttreatment (M = 24.85, SD = 3.22; *p* = 0.524). However, a significant decrease in waist circumference was observed, with the mean score dropping from baseline (M = 83.89, SD = 7.52) to posttreatment (M = 82.77, SD = 8.15; *p* = 0.022). No significant change was found in the waist‐to‐hip ratio, with baseline (M = 0.88, SD = 0.05) and posttreatment (M = 0.87, SD = 0.05) values (*p* = 0.115).

Both sides showed significant improvements from baseline across all sessions (*p* < 0.001). On Side A, the median GAIS score increased from 0 (IQR = 0–1) at the first session to 1 (IQR = 1–1) by the final session. On Side B, the median score started at 0 (IQR = 0–1) in the first session and increased to 2 (IQR = 1–2) at the completion.

Differences in PS scores were evaluated between baseline (assigned a value of 0 for both sides) and each session on both Side A and Side B. Significant differences from the baseline were observed across all sessions on both sides (*p* < 0.001). On Side A, the PS score increased from a median of 2 (IQR = 1–3) in the first session to 3 (IQR = 3–4) by Session 3, remaining consistent at 3 (IQR = 3–4) through the completion of the study. On Side B, the median PS score was 2 (IQR = 1–2.25) at the first session and increased to 4 (IQR = 4–4) by the final session.

Correlation analysis between patient profile variables and posttreatment improvements in WHR, GAIS, and PS revealed no significant associations for age, parity, months postdelivery, FST, or mode of delivery. Notably, a moderate correlation between MET score and GAIS (*p* = 0.413, *p* = 0.019) indicates that higher levels of physical activity, such as jogging for at least 30 min per day, five times per week, or engaging in moderate‐intensity activities like brisk walking or cycling for a similar duration were associated with higher GAIS scores.

There was no significant correlation between WHR and GAIS scores (*p* = 0.003, *p* = 0.858), WHR and PS scores (*p* = 0.063, *p* = 0.732), or between GAIS and PS scores (*p* = −0.081, *p* = 0.659). All associations were found to be nonsignificant (*p* > 0.05), suggesting that posttreatment WHR results were unrelated to GAIS or PS scores. No clear relationship was identified between GAIS and PS scores.

Comparison of the GAIS scores between Side A and Side B per session, as shown in Table 4, showed that there were no significant differences in the first, second, or third microwave sessions, with both sides showing similar median scores (*p* values > 0.802). However, after the CO_2_ laser session, Side B showed a significant improvement (median = 2, IQR = 1–2) compared to Side A (median = 1, IQR = 1–1), with a *p* value of < 0.001, highlighting a notable enhancement on Side B.

**TABLE 4:** Comparison of global aesthetic improvement scale (GAIS) scores between Side A and Side B per session.

Follow‐up	Side A		Side B		*p*
	Median	IQR	Median	IQR	
First microwave session	0	(0–1)	0	(0–1)	1.000
Second microwave session	1	(0–1)	1	(0–1)	0.802
Third microwave session	1	(0.75–1)	1	(0.75–1)	1.000
CO_2_ laser session (Side B only)	1	(1–1)	2	(1–2)	**< 0.001**

Comparison of the PS scores between Side A and Side B across sessions, as detailed in Table 5, revealed that there were no significant differences in PS scores during the first, second, and third microwave sessions, in which both sides had similar median scores (*p* > 0.899). However, following the CO_2_ laser session, Side B showed a significantly higher PS score (median = 4, IQR = 4–4) compared to Side A (median = 3, IQR = 3–4), with a *p* value of 0.001, indicating a significant improvement on Side B.

**TABLE 5:** Comparison of patient satisfaction (PS) scores between Side A and Side B per session.

Follow‐up	Side A		Side B		*p*
	Median	IQR	Median	IQR	
First microwave session	2	(1–3)	2	(1–2.25)	0.899
Second microwave session	2	(2–3)	2	(2–3)	1.000
Third microwave session	3	(3–4)	3	(3–4)	1.000
CO_2_ laser session (Side B only)	3	(3–4)	4	(4–4)	**0.001**

**FIGURE 1:**
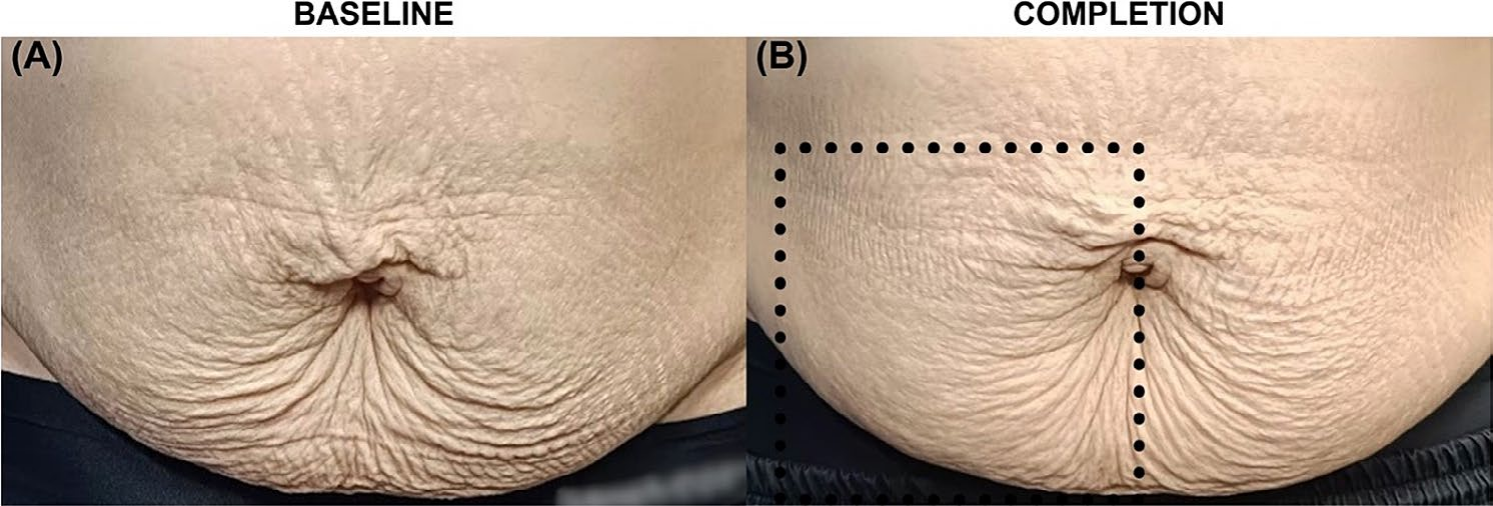
Clinical improvement of the periumbilical and lower abdominal skin laxity in a 42‐year‐old subject with FST IV who had delivered full‐term thrice vaginally and is 7 years postpartum, from (A) baseline to (B) completion follow‐up (CO_2_ laser was done within the dotted area). The patient maintained a weight of 55 kg throughout the study.

Figure 1 illustrates the visible improvement in abdominal skin laxity in a representative patient throughout the treatment sessions, characterized by reduced sagging in the periumbilical region and decreased wrinkling of the hypogastric skin. Notably, more pronounced improvement was evident on Side B (the right side).

**FIGURE 2:**
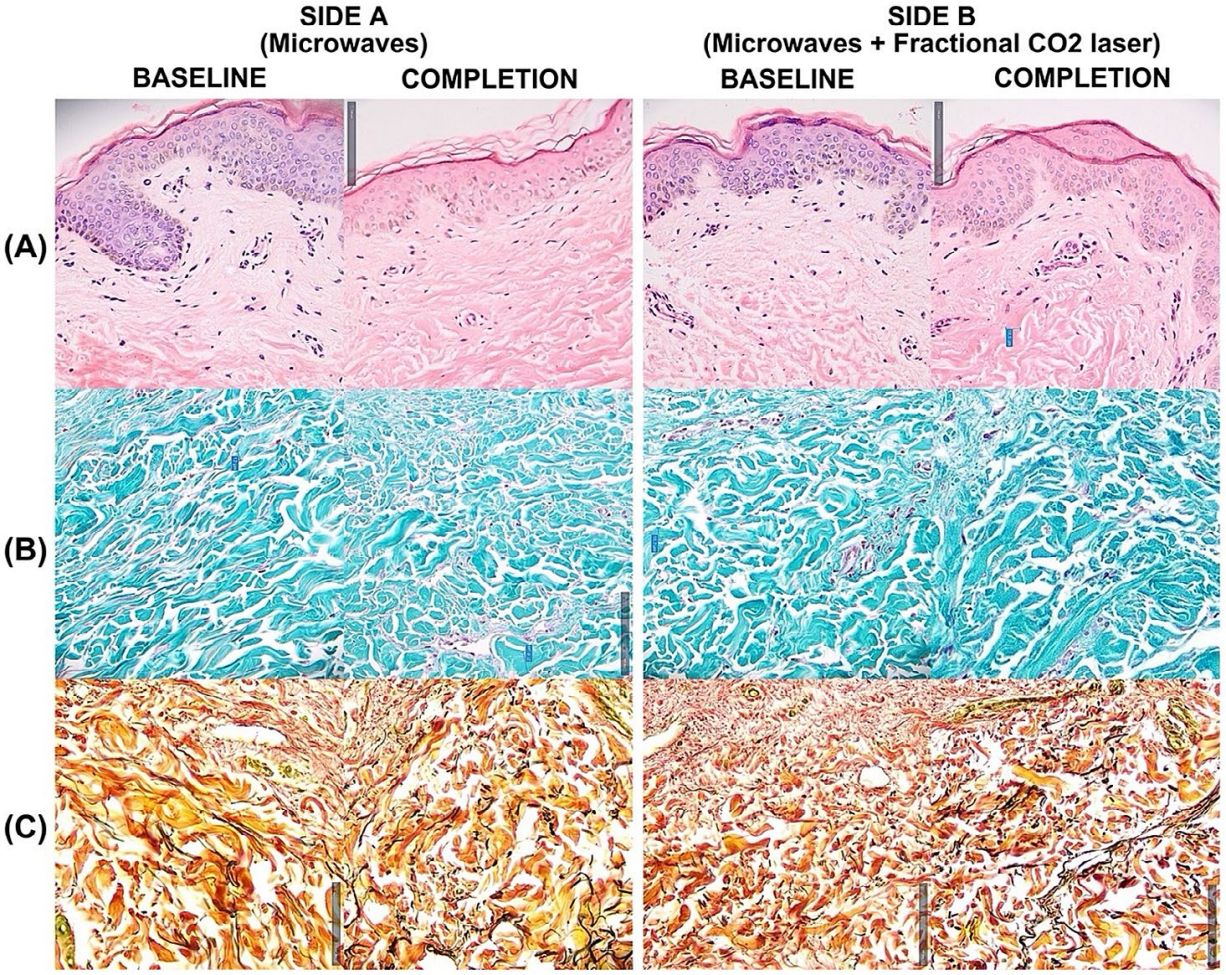
Histological changes of the skin in Side A and Side B on × 200 magnification in a 42‐year‐old subject who had delivered full‐term thrice vaginally and is 7 years postpartum. No visible change in epidermal thickness and dermal collagen density was noted on (A) hematoxylin and eosin and (B) Masson Trichrome stain (collagen bundles are stained blue); however (C) Verhoeff–Van Gieson stain demonstrated a higher density of elastin fibers (stained black), more prominent on Side B.

Skin punch biopsy was done on this patient, which revealed a visible increase in the elastic fiber density on both sides of the abdomen, more pronounced on Side B, as shown in Figure 2.

Adverse effects occurred exclusively during laser treatment in all subjects. Erythema and pain were the most common, affecting all 32 cases (100%). Pruritus was noted in 25 cases (78%) and hyperpigmentation, the least frequent, occurred in seven cases (22%). Most patients tolerated these adverse effects with no management done aside from twice‐daily application of mupirocin 2% ointment and as‐needed use of a cool compress. Hyperpigmentation was sufficiently addressed with twice‐daily use of niacinamide 4% cream until resolution.

## 4 Discussion

Abdominal laxity, commonly referred to as “sagging skin,” arises from a reduction in key dermal components such as collagen, elastin, and hyaluronic acid and is distinct from elasticity (the skin's ability to recoil) and crepiness (fine wrinkling) [13]. In postpartum women, laxity manifests as redundant skin around the waist, lower abdomen, and cesarean section scars [2], bound superiorly by the umbilicus, laterally by the anterior superior iliac spines, and inferiorly by the symphysis pubis [14]. The condition is exacerbated by aging and photodamage, resulting in further collagen degradation [13].

Despite achieving prepregnancy weight, postpartum abdominal laxity is persistent and can be emotionally and psychologically distressing [3]. This issue is further compounded by the growing trend of later pregnancies, increasing multiple births, and societal emphasis on body contouring [15]. As patients age, progressive collagen loss in the abdominal wall results in a more pronounced expansion of skin during pregnancy [16]. Mechanical stress and elastin degradation also contribute to the widening and distortion of the rectus abdominis muscles, fat accumulation, and skin laxity [16].

Traditional treatment options for postpartum abdominal laxity include abdominoplasty, which remains the gold standard, providing immediate and significant results [16]. However, advancements in technology have led to a surge in noninvasive skin‐tightening modalities. These include microfocused ultrasound (MFU‐V), monopolar radiofrequency (RF), subdermal monopolar RF (SMRF), microneedling, and dermal fillers [16]. While less effective than surgery, these methods are increasingly preferred due to their lower risk, minimal recovery time, and reduced costs [17].

One such emerging modality is microwave technology, which utilizes 2.45 GHz microwave radiation to target dermal and subdermal structures [18]. Initially used for cancer ablation [19], its application in body contouring is more recent, addressing localized fat, cellulite, and skin laxity [18]. Microwaves tighten skin by heating the dermal and subdermal layers to 65°C–75°C, leading to collagen denaturation, recoil, and long‐term remodeling [4].

CO_2_ lasers are another effective treatment for skin laxity, which use a 10,600‐nm infrared wavelength to vaporize tissue and stimulate neocollagenesis [20]. Though ablative in nature, resulting in expected side effects like erythema, discomfort, and pigmentation [21], fractional CO2 lasers provide a more tolerable approach by delivering energy in microthermal zones [22]. This allows deeper dermal targeting while leaving surrounding skin intact, enabling viable transient amplifying cells to rapidly reepithelialize the treated areas while reducing the risk of scarring and minimizing downtime [22]. The efficacy of this modality in treating facial skin laxity has been extensively studied, but limited evidence exists regarding its use for larger areas with fewer pilosebaceous units, such as the abdomen [16]. However, its mechanism of action on the more superficial layers of the skin may augment the action of nonablative modalities, which act deeper in the dermis and the subcutaneous layer [16], such as the use of microwaves.

To our knowledge, no studies have been published in indexed literature documenting the histological effects of microwave therapy and fractional CO_2_ laser on the layers of the abdominal skin, following a search of PubMed, Scopus, and Google Scholar using relevant terms.

Currently, no gold standard exists for noninvasive treatments of postpartum abdominal laxity. While surgical options like abdominoplasty remain the most effective, combining noninvasive modalities such as microwave technology and fractional CO2 lasers may offer a balanced approach for patients unwilling to undergo surgery [16]. Further clinical trials are necessary to establish a standardized treatment approach [16].

Laxity can be evaluated in three attributes that are visible (sagging), topographic (roughness), and mechanical (stretchability) [13]. This study evaluated changes using the former two, comparing baseline and follow‐up GAIS scores (given by third‐party assessors using standardized photographs) and PS scores. To the best of our knowledge, no validated tools exist for grading abdominal laxity.

This is the first study to compare the efficacy and safety of the combined approach of the microwave system and fractional CO_2_ laser versus microwaves alone in the treatment of postpartum abdominal skin laxity. Thirty‐two Filipino postpartum patients with a mean age of 40.78 and parity of 2.03, predominantly FST IV (53.13%), completed this study.

Bonan and Verdelli [12], conducted a pilot study on 15 postpartum women, administering three sessions of microwave therapy and an additional one fractional CO2 laser, with 1 month in between sessions. PS scores were the subjective outcome parameter, and WC, WHR, BMI, blood tests, and quantitative volume changes using three‐dimensional (3D) stereo‐photogrammetry were the objective outcome parameters. They found that 46.7% of participants were very satisfied while 53.3% were satisfied and that the procedure was well‐tolerated by the participants, with no reported side effects. All objective parameters showed significant improvement (*p* < 0.001) on follow‐up, and overall, there was a great improvement in skin laxity. Their results were congruent with ours, in which patients were generally satisfied with their improvement after 1 month, with increasing levels of satisfaction per additional microwave session. Compared to three sessions of microwave alone, the addition of a CO_2_ laser session resulted in higher patient satisfaction, mostly extremely satisfied, despite adverse effects such as erythema, pain, pruritus, and hyperpigmentation. Since patient satisfaction is a highly subjective and complex metric, influenced by preprocedural expectations [23] and unmodifiable patient‐level characteristics [24], to add an objective parameter, this study also determined clinical improvement (sagging, wrinkling) by a GAIS scoring, validated in repeatability, administered by blinded experts in aesthetic dermatology who assessed baseline and posttreatment standardized photographs. Although there was no significant correlation between PS and GAIS scores, it was found that the assessors noted visible improvement across all sessions, with significantly higher GAIS scores indicating marked improvement of the appearance, but not completely optimal, on Side B compared to baseline.

At least one treatment is said to yield visible results for both the microwaves system and CO2 laser, often seen at least 1 month after the session [18, 22], and this was observed in all our patients.

Bonan and Verdelli [12] reported a significant reduction in WC (M = 3.6, SD = 1.2), similar to our findings, though this did not significantly affect WHR or BMI. Notably, reductions in these parameters are not expected with the skin tightening function of the microwave system, which targets part of the dermis and subdermis. They additionally reported near‐universal repositioning of the umbilicus to its original anatomical location; a finding not detected in our patients. This may be due to their extended follow‐up period of 8 weeks posttreatment and their use of 3D‐stereophotogrammetry, which allows for a highly accurate evaluation of body landmarks [25]. Both studies observed improvements in striae distensae, likely due to dermal collagen remodeling from microwaves and CO_2_ laser treatment, though quantifying the extent of this improvement was beyond the scope of our study.

A significant correlation between physical activity and GAIS scores was noted. Kjaer [26] suggests that physical activity influences extracellular matrix turnover by enhancing growth factor release, transcription, and posttranslational modifications. Although no studies have examined the effects of exercise on dermal collagen remodeling, research on muscles and tendons in young resistance‐trained males [27] shows increased local blood flow, IL‐6 [28], and matrix metalloproteinases, promoting collagen turnover and synthesis. However, these findings are not necessarily applicable to skin or postpartum women.

Postpartum abdominal skin laxity is histologically characterized by epidermal thinning [29], reduced dermal collagen and elastin [13], and fewer, thinner fibrous septae in the subdermis [14]. Skin biopsies at baseline and completion revealed increased elastin density in Side A and Side B, demonstrating the capacity of the microwave system to rebuild the elastic scaffold of the dermis. This improvement was more pronounced on Side B, showing that CO_2_ laser enhances this effect. No subdermal tissue was sampled. Increase in collagen density and epidermal thickness was not observed, although it may be seen with additional treatment sessions and tissue samples from other patients.

Adverse effects of the microwave treatment are typically minimal, including transient erythema, edema, bruising, burns, bullae, adipose atrophy, and hyperpigmentation [12]. In this study, no such effects were observed. Adverse reactions occurred only after CO_2_ laser treatment, with erythema lasting less than a week and pain subsiding within 2–3 days. Some patients reported pruritus and hyperpigmentation, lasting less than 7 days and 22 days on average, respectively. Severity scores remained below 4 out of 10, indicating the intervention was safe and well tolerated.

## 5 Conclusion

The combined use of the microwave system and fractional CO_2_ laser is safe and well tolerated and is superior to microwaves alone in the treatment of postpartum abdominal laxity.

## 6 Limitations of the Study

The account of relapse or further improvement after 1 month posttreatment completion was not determined. The degree of improvement of striae or adiposity was not quantified. Results will not reflect on areas other than the abdomen. Histopathologic findings were obtained from only one patient, without evaluation of the subdermis, and are not generalizable. Objective assessments were done with an assessor‐administered scoring of standardized photographs without the measurement of skin elasticity. Though other skin laxity grading systems exist for areas such as the face, neck, buttocks, and thighs, validated scales for abdominal skin laxity remain unavailable. Thus, GAIS scoring was employed as a practical and repeatable alternative.

## 7 Recommendations

The duration of skin laxity in patients may be determined. Subjects with a smoking history, as well as data on dietary intake, could be included in future studies to investigate whether these factors can affect outcomes. While this study utilized cold compress application, which is a widely used method for reducing postlaser side effects, adjunctive therapies such as platelet‐rich plasma (PRP) may be used instead to further enhance recovery and reduce adverse effects without compromising the thermal effect from microthermal zones necessary for dermal remodeling. The use of a skin analyzer, ultrasound skin imaging, and 3D stereo‐photogrammetry may provide high‐quality objective insights acquired in a noninvasive manner to evaluate improvements in abdominal laxity. Quantifying histologic improvements up to the subcutaneous fat of all enrolled subjects may yield more precise and generalizable data. Future studies may employ longer follow‐up periods to assess long‐term efficacy. Studies on the efficacy of the microwave system alone versus its combined use with other modalities in the treatment of other aesthetic concerns such as striae distensae, cellulite, and localized fat have not been explored, and are areas of interest.

## Conflicts of Interest

The authors declare no conflicts of interest.

## Supporting information


Data S1.

## Data Availability

The data that support the findings of this study are openly available in Mendeley Data powered by Digital Commons Data repository. at https://data.mendeley.com/datasets/sh82cm2x64/1, reference number 10.17632/sh82cm2x64.1.
