# Supplementary material for: Microwaves Versus Combined Microwaves and Fractional Carbon Dioxide Laser in the Treatment of Postpartum Abdominal Laxity Among Filipino Patients in a Tertiary Hospital: A Randomized, Prospective, Assessor‐Blinded, Simultaneous Split‐Abdomen Trial
Source: J Cosmet Dermatol. 2025 May 15;24(5):e70237. doi: 10.1111/jocd.70237 (PMC12079721; doi:10.1111/jocd.70237)
Supplement: Supplementary file 1 — Data S1. [file JOCD-24-e70237-s001.docx]

| **PROFILE** | | | | | | | | | | | | |
| --- | --- | --- | --- | --- | --- | --- | --- | --- | --- | --- | --- | --- |
| **Patient Code:** | | **Date Enrolled:** | | | | | | | | | | |
| **Age:** | | **Fitzpatrick skin type: III IV V** | | | | | | | | | | |
| **Parity: G___ P___** | | **Number of months post-delivery:** | | | | | | | | | | |
| **Type of delivery: Vaginal Abdominal** | | | | | | | | | | | | |
| **Level of Physical Activity**   \| **Work/**  **occupation** \| **Walking/**  **bicycling** \| **Home/**  **household work** \| **Watching TV/**  **reading** \| **Exercise** \| \| --- \| --- \| --- \| --- \| --- \| \| **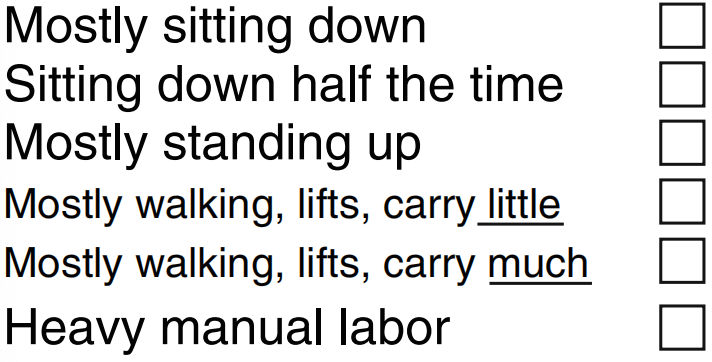** \| **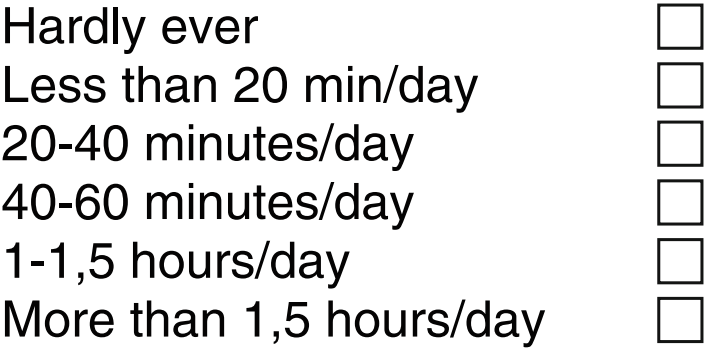** \| 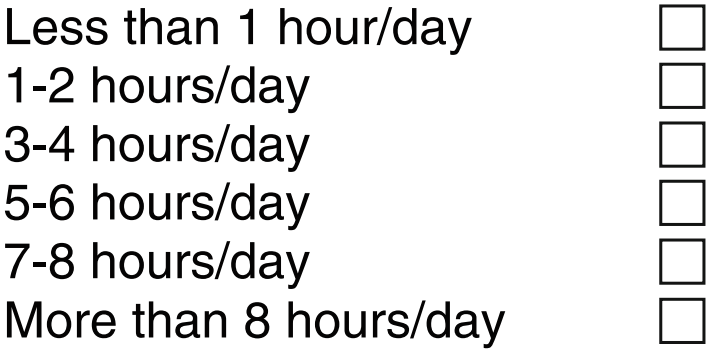 \| **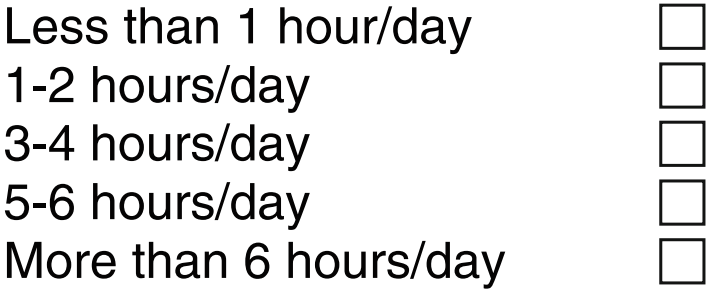** \| **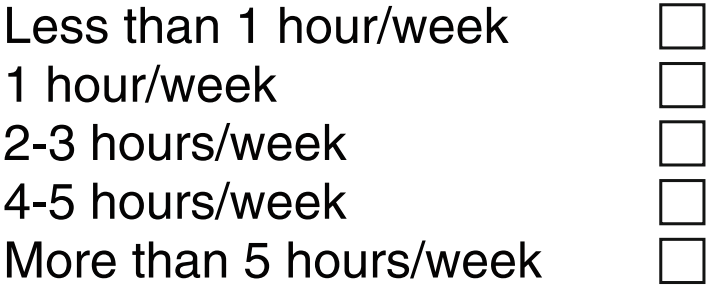** \| \| **METs:** \| **METs:** \| **METs:** \| **METs:** \| **METs:** \| \| **Hours of Sleep:**  **METs:** \| \| **Total METs/day:** \| \| \| | | | | | | | | | | | | |
| **BASELINE DATA** | | | | | | | | | | | | |
| **Height (m):** | | | | **Weight (kg):** | | | | **BMI:** | | | | |
| **WC (cm):** | | | | **HC (cm):** | | | | **WHR (cm):** | | | | |
| **1^ST^ MICROWAVES SESSION** | | | | | | | | | | | | |
| **Adverse effects: None** | | | | | | | | | | | | |
| **Sign/Symptom** | **Laterality** | | **Severity** | | | | **Onset** | | **Duration** | | | **Management** |
|  |  | |  | | | |  | |  | | |  |
|  |  | |  | | | |  | |  | | |  |
|  |  | |  | | | |  | |  | | |  |
| **Follow-up Date:** | | | **L** | | **GAIS scores:** | | |  | |  |  | **PS score:** |
|  |  |  | **R** | | **GAIS scores:** | | |  | |  |  | **PS score:** |
| **2^ND^ MICROWAVES SESSION** | | | | | | | | | | | | |
| **Adverse effects: None** | | | | | | | | | | | | |
| **Sign/Symptom** | **Laterality** | | **Severity** | | | | **Onset** | | **Duration** | | | **Management** |
|  |  | |  | | | |  | |  | | |  |
|  |  | |  | | | |  | |  | | |  |
|  |  | |  | | | |  | |  | | |  |
| **Follow-up Date:** | | | **L** | | **GAIS scores:** | | |  | |  |  | **PS score:** |
|  |  |  | **R** | | **GAIS scores:** | | |  | |  |  | **PS score:** |
| **3^RD^ MICROWAVES SESSION** | | | | | | | | | | | | |
| **Adverse effects: None** | | | | | | | | | | | | |
| **Sign/Symptom** | **Laterality** | | **Severity** | | | | **Onset** | | **Duration** | | | **Management** |
|  |  | |  | | | |  | |  | | |  |
|  |  | |  | | | |  | |  | | |  |
|  |  | |  | | | |  | |  | | |  |
| **Follow-up Date:** | | | **L** | | **GAIS scores:** | | |  | |  |  | **PS score:** |
|  |  |  | **R** | | **GAIS scores:** | | |  | |  |  | **PS score:** |
| **FRACTIONAL CO2 LASER SESSION (TREATMENT SIDE ONLY)** | | | | | | | | | | | | |
| **Treatment side: Left Right** | | | | | | | | | | | | |
| **Adverse effects: None** | | | | | | | | | | | | |
| **Sign/Symptom** | **Laterality** | | **Severity** | | | | **Onset** | | **Duration** | | | **Management** |
|  |  | |  | | | |  | |  | | |  |
|  |  | |  | | | |  | |  | | |  |
|  |  | |  | | | |  | |  | | |  |
| **Follow-up Date:** | | | **L** | | **GAIS scores:** | | |  | |  |  | **PS score:** |
|  |  |  | **R** | | **GAIS scores:** | | |  | |  |  | **PS score:** |
| **1 MONTH POST-TREATMENT COMPLETION DATA** | | | | | | | | | | | | |
| **Height (m):** | | | | | | **Weight (kg):** | | | | **BMI:** | | |
| **WC (cm):** | | | | | | **HC (cm):** | | | | **WHR (cm):** | | |
